# Quantitation of dynamic total-body PET imaging: recent developments and future perspectives

**DOI:** 10.1007/s00259-023-06299-w

**Published:** 2023-07-18

**Authors:** Fengyun Gu, Qi Wu

**Affiliations:** 1 School of Mathematics and Physics, North China Electric Power University, 102206 Beijing, China; 2 School of Mathematical Sciences, University College Cork, T12XF62 Cork, Ireland

**Keywords:** Total-body PET, Multiple organs, Arterial input function, Kinetic models, Parametric imaging

## Abstract

**Background:**

Positron emission tomography (PET) scanning is an important diagnostic imaging technique used in disease diagnosis, therapy planning, treatment monitoring, and medical research. The standardized uptake value (SUV) obtained at a single time frame has been widely employed in clinical practice. Well beyond this simple static measure, more detailed metabolic information can be recovered from dynamic PET scans, followed by the recovery of arterial input function and application of appropriate tracer kinetic models. Many efforts have been devoted to the development of quantitative techniques over the last couple of decades.

**Challenges:**

The advent of new-generation total-body PET scanners characterized by ultra-high sensitivity and long axial field of view, i.e., uEXPLORER (United Imaging Healthcare), PennPET Explorer (University of Pennsylvania), and Biograph Vision Quadra (Siemens Healthineers), further stimulates valuable inspiration to derive kinetics for multiple organs simultaneously. But some emerging issues also need to be addressed, e.g., the large-scale data size and organ-specific physiology. The direct implementation of classical methods for total-body PET imaging without proper validation may lead to less accurate results.

**Conclusions:**

In this contribution, the published dynamic total-body PET datasets are outlined, and several challenges/opportunities for quantitation of such types of studies are presented. An overview of the basic equation, calculation of input function (based on blood sampling, image, population or mathematical model), and kinetic analysis encompassing parametric (compartmental model, graphical plot and spectral analysis) and non-parametric (B-spline and piece-wise basis elements) approaches is provided. The discussion mainly focuses on the feasibilities, recent developments, and future perspectives of these methodologies for a diverse-tissue environment.

## Introduction

In recent years, positron emission tomography (PET) has a wide range of clinical and research applications in oncology, cardiology, and neurology [1, 2]. It is a unique imaging modality that enables the measurements of a diverse range of functional and biological processes (e.g., tumor metabolism [3], proliferation [4], blood flow [5], and receptor-binding [6]), depending on the administrated radiotracer. In daily clinical practice, PET imaging is obtained at a single time point and assessed visually or using simple indices, e.g., standardized uptake value (SUV) [7]. Although these are sufficient for many diagnostic applications, dynamic scans with multiple time frames are implemented in some research avenues for advanced diagnosis, response assessment, therapy management, and drug/tracer development [8, 9].

Since the 1950s, there have been great advances with PET including the introduction of time-of-flight technologies [10], optimized detectors [11, 12], new radiotracers [13], iterative reconstruction algorithms [14, 15], and novel quantitative methods [16, 17] by a variety of scientists in physics, engineering, chemistry, mathematics, and statistics [18–20]. However, some constraints such as the limited axial coverage still exist [21]. Currently, the conventional PET/CT systems have a short axial field of view (AFOV) of about $15\sim 30$ cm and typically only a specific organ such as the brain is imaged. On these scanners, whole-body (even dynamic) scanning can be performed by a multi-bed scenario, but pitfalls like the missing early-phase data and low temporal resolution limit its wide use [22].

The revolutionary total-body (TB) PET scanners (e.g., uEXPLORER [23], PennPET Explorer [24], and Siemens Biograph Vision Quadra [25]) have been developed to overcome these limitations. Such devices enable the simultaneous image of entire human body or main organs using a single-bed position. Given their ultra-high sensitivity (10$\sim$40 fold), extended field of view (1$\sim$2 m), and enhanced temporal resolution (20$\sim$200 time frames), the potential clinical applications of these innovative technologies have been exploited in different ways to provide better image quality [25–28], reduce scan time [29–34], lower the injected dose [35–38], and develop new drugs; see [21, 39–44] for more descriptions. Next to all the exciting opportunities that arise with TB systems, there remain some challenges. The analysis of large-scale data, especially for dynamic scanning, becomes one.

Quantitation of dynamic PET studies could be able to provide additional biological information, and the potential benefits have been highlighted in precision medicine [45–47]. A broad family of quantitative techniques with focus on the recovery of arterial input function and the establishment of tracer kinetic model has been proposed to estimate the kinetic parameters of interest. The other procedures including motion correction and denoising also have some impacts on the estimated kinetics. Many different points of view have been taken in extensive literature and more comprehensive references [8, 16, 17, 48–52] are suggested for further readings. The aim of this review is to provide an overview of the basic principles and model formulations of the most important strategies for PET quantitation, along with their feasibilities and recent developments for the emerging total-body PET imaging. The future perspectives to further enhance quantitative accuracy are discussed as well.

## Total-body PET studies

**Fig. 1:**
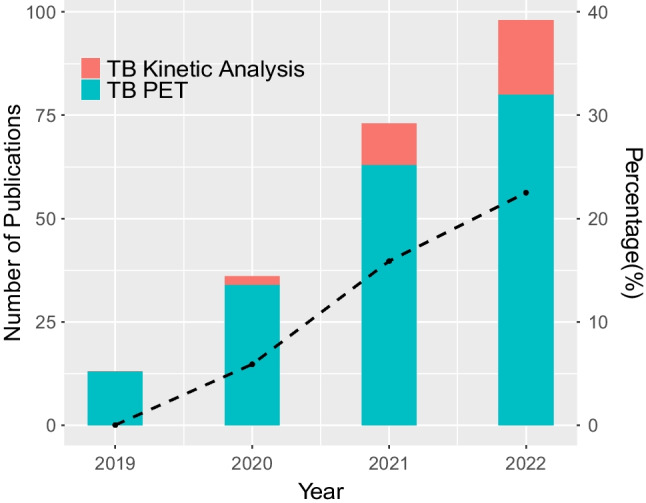
The number of publications (left *y*-axis) on the total-body (TB) PET studies (blue) and dynamic TB scanning with the implementation of kinetic analysis (red) for the period from 2019 to 2022. The percentage (right *y*-axis) of publications relevant to the kinetic model in TB PET is shown as the black line. The data were collected from a search on PubMed on 08/05/2023

Since the first total-body human imaging was obtained on the uEXPLORER scanner in Zhongshan hospital [23], the spread of uEXPLORER with other long axial field of view ($>1$m) systems has become worldwide. Up to 2022, more than 10 total-body PET/CT scanners, including uEXPLORER, PennPET Explorer, and Biograph Vision Quadra, have been installed in China [53], the USA [24, 54], and Europe [25, 38, 55]. The use of such scanners in both clinical (static mode) and research (dynamic mode) settings is emerging. Figure 1 shows the trend for the work in the area of total-body PET from 2019 to 2022. The number of publications has a significant increase and reached to approximate 200 over the past 4 years. The proportion of dynamic studies with the implementation of kinetic analysis in total-body PET also steadily increases each year.

A list of reported dynamic total-body PET study cohorts along with the specific details is provided in Table 1. Several types of subjects were recruited: healthy volunteers and patients diagnosed with cancer or infected with COVID-19. While the most of scans were done exclusively with the administration of fluorine-18 labeled fluorodeoxyglucose (${}^{18}$F-FDG), there are other radiotracers of interest to be employed, such as ${}^{68}$Ga-FAPI-04 [56–58], ${}^{15}$O-H${}_2$O [59], ${}^{89}$Zr-Df-Crefmirlimab [60, 61], ${}^{18}$F-Fluciclovine [62], and [${}^{11}$C]methionine [63]. A range of scanning and reconstruction protocols have been applied by different hospitals/institutions, but the magnitude of image voxels is generally on the order of ten million, and a more dense sequence is commonly performed at the early time. Although these dynamic datasets may not be consistent, the data analysis should face similar problems that will be discussed carefully in the next section.

**Table 1:** List of reported dynamic studies on total-body PET scanners (uEXPLORER, PennPET Explorer, and Biograph Vision Quadra)

PET scanner	Radiotracer	No	(Voxels, time-frames)	Subject type	Site	Temporal sequences	Ref
uEXPLORER	${}^{18}$F-FDG	1	$(236\times 236\times 679,187)$	Healthy	[a]	$60\times 1s,30\times 2s,20\times 3s,12\times 10s,50\times 30s,15\times 120s$	[64]
	${}^{18}$F-FDG	11	$(236\times 236\times 679,97)$	Healthy	[a]	$24\times 5s,73\times 60s$	[29]
	${}^{18}$F-FDG	30	$(236\times 236\times 679,60)$	Healthy	[a]	$36\times 5s,24\times 180s$	[35, 65, 66]
	${}^{18}$F-FDG	35	$(236\times 236\times 679,55)$	Cancer	[a]	$36\times 5s,19\times 180s$	[67]
	[${}^{68}$Ga]Ga-DOTA-FAPI-04	19	$(192\times 192\times$ NA,NA)	Malignancy	[a]		[58]
	[${}^{68}$Ga]Ga-DOTA-TATE	7	$(192\times 192\times$ NA,55)	Cancer	[a]	$36\times 5s,19\times 180s$	[68]
	${}^{18}$F-FDG	7	$(192\times 192\times 672,70)$	Cancer	[b]	$30\times 5s,15\times 30s,25\times 120s$	[69]
	${}^{18}$F-FDG	28	$(192\times 192\times 672,66)$	Healthy/cancer	[b]	$24\times 5s,6\times 10s,6\times 30s,6\times 60s,24\times 120s$	[70]
	${}^{18}$F-FDG	15	$(192\times 192\times 673,25)$	Cancer	[b]	$1\times 30s,3\times 10s,4\times 30s,5\times 60s,4\times 180s,8\times 300s$	[31]
	${}^{18}$F-FDG	200	$(192\times 192\times 673,98)$	Healthy/cancer	[b]	$50\times 2s,20\times 10s,10\times 30s,10\times 60s,8\times 300s$	[71]
	${}^{18}$F-FDG	13	$(150\times 150\times 486,120)$	Healthy	[c]	$60\times 1s,30\times 2s,6\times 10s,6\times 30s,12\times 120s,6\times 300s$	[72]
	${}^{18}$F-FDG	21	$(150\times 150\times 486,66)$	Healthy/cancer	[c]	$30\times 2s,12\times 10s,6\times 30s,12\times 120s,6\times 300s$	[73]
	${}^{18}$F-FDG	10	$(150\times 150\times 486,29)$	Healthy/cancer	[d]	$6\times 10s,2\times 30s,6\times 60s,5\times 120s,4\times 180s,6\times 300s$	[54]
	${}^{18}$F-FDG	7	(NA,120)	COVID-19	[d]	$60\times 1s,30\times 2s,6\times 10s,6\times 30s,12\times 120s,6\times 300s$	[74]
	${}^{11}$C-Butanol	3	(NA,29)	Healthy/peripheral artery disease	[d]	$12\times 5s,6\times 10s,6\times 30s,5\times 300s$	[75]
	${}^{89}$Zr-Df-Crefmirlimab	8	$(512\times 512\times$ NA,46)	Healthy/COVID-19	[d]	$6\times 60s,16\times 30s,2\times 60s,12\times 120s,10\times 300s$	[60, 61]
	${}^{18}$F-Fluciclovine	37	$(256\times 256\times$ NA, NA)	Cancer	[d]		[62]
	${}^{18}$F-FDG	30	$(360\times 360\times 672,92)$	Cancer	[e]	$30\times 2s,12\times 5s,6\times 10s,4\times 30s,25\times 60s,15\times 120s$	[76]
	${}^{68}$Ga-FAPI-04	9	$(239\times 239\times 679,92)$	Cancer	[f]	$30\times 2s,12\times 5s,6\times 10s,4\times 30s,25\times 60s,15\times 120s$	[56]
	${}^{68}$Ga-FAPI-04	13	$(360\times 360\times$NA,92)	Cancer	[f]	$30\times 2s,12\times 5s,6\times 10s,4\times 30s,25\times 60s,15\times 120s$	[57]
	[${}^{11}$C]methionine	12	(NA,67)	Multiple myeloma (MM)	[f]	$30\times 2s,12\times 5s,6\times 10s,4\times 30s,15\times 60s$	[63]
PennPET Explorer	${}^{18}$F-FDG	4		Healthy/cancer	[g]		[24, 77]
Biograph Vision Quadra	${}^{18}$F-FDG	12	$(220\times 220\times 708,31)$	Cancer	[h]	$6\times 10s,3\times 20s,6\times 30s,5\times 60s,11\times 300s$	[55]
	${}^{18}$F-FDG	24	$(440\times 440\times 645,62)$	Cancer	[i]	$2\times 10s,30\times 2s,4\times 10s,8\times 30s,4\times 60s,5\times 120s,9\times 300s$	[25, 78, 79]
	[${}^{82}$Rb]C1	1		Atypical chest pain	[i]		[80]
	${}^{15}$O-H$_2$O	5	$(440\times 440\times 645,54)$	Cancer	[j]	$1\times 5s,30\times 1s,15\times 2s,5\times 10s,3\times 20s$	[59]

The corresponding radiotracer, the number of patients (No.), data dimension (voxels, time-frames), subject type, site of scanning and temporal sequences in each cohort are presented

[*a*] Zhongshan Hospital, Fudan University, Shanghai, China

[*b*] Henan Provincial People’s Hospital People’s Hospital of Zhengzhou University, Henan, China

[*c*] University of California Davis, California, USA

[*d*] University of California Davis EXPLORER Molecular Imaging Center, California, USA

[*e*] Sun Yat-Sen University Cancer Center, Guangzhou, China

[*f*] Renji Hospital, Shanghai, China

[*g*] University of Pennsylvania, Philadelphia, USA

[*h*] University of Groningen, Groningen, The Netherlands

[*i*] Bern University Hospital, University of Bern, Bern, Switzerland

[*j*] University of Copenhagen, Denmark

## Opportunities and challenges in dynamic total-body PET imaging

As summarized in Table 2, the unique characteristics of total-body PET studies bring a series of new challenges and opportunities for improved quantitative accuracy. Details are presented below:

(i) Improved image-derived input function: Due to the long axial field of view of total-body PET scanners, image-derived input functions can be measured from multiple blood pools (e.g., heart ventricle, aorta, and artery). Higher temporal resolution (e.g., 1 s even 0.1s per early frame) also allows better temporal sampling of the extracted input function [43, 64, 81].
(ii) Organ-dependent time delay: The arrival time of tracer to different organs is significantly varied, which has been an important factor for accurate total-body kinetics [54, 82–84].
(iii) Tissue-specific kinetics: Each kind of tissue has its own physiological mechanism and some tissues such as the liver, kidney, and bladder even exhibit more complex kinetics. Thus, a single kinetic model may not be feasible for multiple organs, and appropriate model selection is necessary [54, 84–86].
(iv) Capture fast kinetics: The high temporal sampling imaging provides an opportunity for better investigation of fast kinetics such as the blood volume or blood flow (perfusion), which are potential biomarkers for the prediction of therapy response or survival [87–90].
(v) High-quality dynamic PET images: The increased sensitivity enables the generation of high signal-to-noise (SNR) images, which is greatly beneficial to the quantitation of dynamic imaging at the voxel level, e.g., noise reduction and lesion enhancement. But we need to note the sensitivity along the axial field of view shows the reciprocal U shape (non-uniform) [28, 91, 92]. Thus, images have higher variances towards the axial edge, which needs to be considered carefully.
(vi) Huge data set: It is challenging to store and process such enormous and complex datasets, which may be addressed by some automation forms using more comprehensive approaches (e.g., segmentation) [93–98] or artificial intelligence [40, 85, 99–101]

**Table 2:** Characteristics and challenges/opportunities of total-body PET scanners

Characteristics	Challenges/opportunities
Multiple organs/tissues	$\bullet$ Tissue-specific kinetics
	$\bullet$ Large blood pool in FOV
	$\bullet$ Heterogeneity
	$\bullet$ Delay correction
Higher temporal resolution	$\bullet$ Capture fast kinetics
Higher spatial resolution	$\bullet$ Better image quality
Huge data set	$\bullet$ High computational cost

**Fig. 2:**
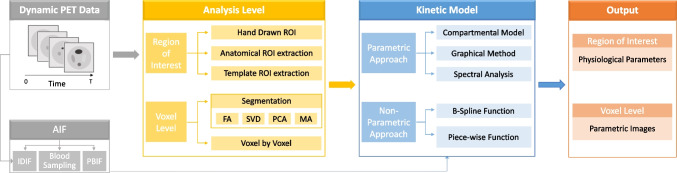
Overview of dynamic PET quantitation. Abbreviations: PET, positron emission tomography; IDIF, image-derived input function; PBIF, population-based input function; ROI, region of interest; FA, factor analysis; SVD, singular value decomposition; PCA, principal component analysis; MA, mixture analysis

## Overview of dynamic PET quantitation

Dynamic PET quantitation is not a single procedure and involves several steps such as the recovery of input function and application of tracer kinetic modeling. The overview of this process is presented in Fig. 2. In the following sections, we will introduce the basic principles and some well-established methodologies, also their further developments for the emerging total-body PET imaging [43, 64, 78].

### Basic equation

Understanding the targeted biochemical pathway is critical for the interpretation of dynamic PET imaging data. It can be approached using the indicator-dilution method built on the seminal work of Meier and Zierler [102]. Assuming the radiotracer’s interaction with tissue is substantially linear and time-invariant (LTI), the vascular network can be regarded as an LTI system with an arterial input. Hence, the measured tissue time activity curve (TAC) - $C_T$ can be expressed as a convolution between the arterial input function ($C_p$) and tissue residue, also called the impulse response function (*R*) as in Eq. 1.

1 $$\begin{aligned} C_{ T }(t)=\int _{ 0 }^{ t }{ R(t-s){ C }_{ p }(s)ds }\equiv R(t) \otimes { C }_{ p }(t) \end{aligned}$$

With the known input function, kinetic analysis is concerned with the estimation of residue and associated kinetic parameters such as flow (*K*), flux ($K_i$) and volume of distribution ($V_D$).

2 $$\begin{aligned} K=R(0), \quad K_i=\underset{t\rightarrow \infty }{lim}R(t), \quad V_D=\int _{0}^{\infty }R\left( t\right) dt \end{aligned}$$

When the model is applied to PET time-course data, there is typically an adjustment for a biologically important parameter—blood volume (${V}_{B}$). Moreover, the site to recover the input function may be remote from the tissue, introducing a time delay. The correction is generally accomplished by the inclusion of a delay term ($\Delta$) in the modeling procedure as Eq. 3. The delay has been found to vary with different voxels/organs/tissues and its correction is necessary [54, 82–84].

3 $$\begin{aligned} { C }_{ T }(t)= { V }_{ B }{ C }_{ p }(t-\Delta )+(1-{ V }_{ B })\int _{ 0 }^{ t }{ R(t-s){ C }_{ p }(s-\Delta )ds } \end{aligned}$$

Some specific organs (e.g., liver) receive dual blood supplies from the hepatic aorta and portal vein [103–106]. To account for such an effect, the input function can be expressed as a weighted sum of both supplies [107–109].

4 $$\begin{aligned} C_p(t)=(1-f_A)C_{PV}(t)+f_AC_A(t) \end{aligned}$$

where $C_{PV}$ is the portal vein input and $C_A(t)$ is the aortic input. $f_A$ is the fraction of hepatic artery to the overall liver blood flow.

### Region of interest versus voxel-level analysis

The computation of kinetic parameters can be performed either at the regional or voxel level. Due to the average of the voxel information in a region of interest (ROI), the noise can be reduced dramatically. ROI analysis leads to more robust results, especially in the case of dynamic PET studies, but also introduces some biases when defining ROIs from a template, summed, or anatomic images [49]. An alternate approach to regional estimates is performing analysis at the voxel level and generated parametric images can reveal the heterogeneity of tumors [16]. However, many issues need to be considered carefully such as computational efficiency, selection of appropriate models, and noise suppression [54].

Total-body PET scanners have the ability to image more organs/tissues using the single-bed position, but the datasets are much bigger and complex than conventional studies. Multivariate statistical methods including factor analysis (FA) [95], singular value decomposition (SVD) [98], principal component analysis (PCA) [94, 97], and mixture analysis (MA) [93] express the dynamic PET data as a weighted sum of image volumes. They enable to identify organs and structures with different kinetic patterns in a temporal sequence and reduce the temporal and spatial variations of the noise [110]. Once the segmentation process is completed, kinetics for each segment TAC (sub-TAC) are calculated and then mapped back to the original spatial space. These data-driven approaches have the great potential to efficiently handle the complexities and address variable noise issues in dynamic total-body images [96].

## Arterial input function

For standard PET quantitation, the knowledge of the tracer arterial plasma concentration is required as an arterial input function (AIF). The input function can be derived either from (i) arterial blood samples, (ii) the time course of an ROI drawn on the PET image, or (iii) based on the population. Here, we provide a brief introduction to these commonly used and model-based approaches together with their applications in total-body PET studies. For more details, readers are referred to two recent review papers [111, 112].

### Blood sampling

Arterial blood sampling during dynamic acquisition has been considered the standard for input function in many references [113–116]. But some concerns are also raised, for example, the measured AIF may suffer from some effects (e.g., delay, dispersion, and metabolites), which need to be corrected [112]. This invasive procedure also implies discomfort for the patient (insertion of arterial lines and increased radiation) and additional costs for the analysis of numerous blood samples. Thus, it is typically used for research purposes and not recommended for routine clinical practice.

Manual blood sampling or an automatic blood sampling system (ABSS) [117] is generally used to collect arterial blood. However, manual separation of plasma requires decay correction [118, 119], while longer tubing in ABSS introduces higher dispersion effects [120] and requires consideration of the blood-to-plasma ratio [121, 122]. Another issue with AIF refers to the metabolite analysis. Although there do not exist blood-based metabolites for some tracers such as ${}^{18}$F-FDG and $^{15}$O-H${}_2$O, most tracers produce isotope-labeled metabolites that contaminate the input function. These metabolites can be corrected by some mathematical models, e.g., hill model [123, 124], power model [125, 126], and exponential models [127]. A review of the commonly used metabolite-correction approaches is suggested to read for further details [128].

In practice, it would be more difficult to get the blood sampling for the total-body PET study. For example, both the radial artery and antecubital vein are harder to access due to the long axial field of view [40]. The long line from the wrist to the sampling site also may cause more serious delay and dispersion issues [112]. With so many challenges, the first attempt was made by a Denmark group to get the arterial blood sample for the total-body ${}^{15}$O-H$_2$O scanning with Quadra [59]. Such clinical trials are expected to be conducted more in the future. On the other hand, some non-invasive techniques (based on image/population/mathematical models) have also been developed as follows.

### Image-derived input function

To obviate the need for blood sampling, input information can be also derived from a region drawn at the blood pool on PET images, referred to as image-derived input function (IDIF). Due to the limited field of view of conventional PET scanners, sometimes IDIF can only be measured from small vessels such as carotids. However, total-body PET imaging provides multiple choices encompassing the left ventricle, aorta, and other big blood vessels [43, 64, 78]. So far, the IDIF recovered from an ROI over descending aorta (DA) has been the most popular one [17]. Furthermore, the high spatial and temporal resolutions may also lead to more accurate and less noisy IDIF.

However, the use of IDIF still needs to be investigated carefully in the total-body setting. The whole blood activity concentration can be derived, and plasma concentration is impossible to obtain. Reliable results are only generated with radiotracers that do not produce any metabolites, such as ${}^{18}$F-FDG [49]. Additional corrections to the IDIF are also important for accurate kinetics [72].

### Population-based input function

Assuming individuals have the same tracer injection protocol and similar physiological characteristics in a cohort, the population-based input function is generally calculated by averaging and scaling this set of input functions using arterial catheterization invasively [129]. Such a method is probably the most interesting approach for use in clinical practice with many radiotracers, but currently, it has been validated almost exclusively for ${}^{18}$F-FDG [130]. Several groups have attempted to reduce the dynamic scanning time using the PBIF on the total-body PET scanners [32, 55, 79].

### Model-based input function

Model-based descriptions of the arterial samples are usually introduced to obtain continuous and noise-free input functions, which may be helpful to further improve IDIF or PBIF. The most famous models are Feng’s model [131] and its variation, i.e., tri-exponential model [132], but they both cannot describe the complex behavior of the AIF and account for different injection protocols properly [133]. Simultaneous estimation of the input function (SIME) is usually used to generate a specific input function by fitting regional TACs simultaneously [134–136]. Recently, a population-based projection model (PBPM) has been developed which combines population profiling (as in a PBIF approach) with individual arterial input data modeling (as in an IDIF approach). This model incorporates knowledge of injection duration into the fit, allowing for varying injection protocols [137]. Another promising model to be applied to the emerging total-body PET imaging is the novel Markov chain model for the representation of the whole-body tracer circulation [138].

**Table 3:** A summary of major kinetic models used in PET quantitation

Kinetic model	Compartmental model	Patlak plot	Spectral analysis	Non-parametric analysis
Graphical representation	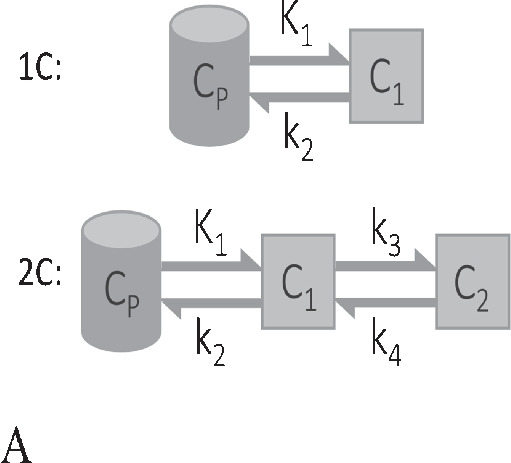	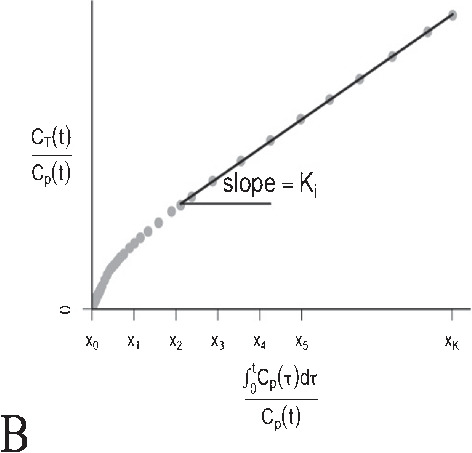	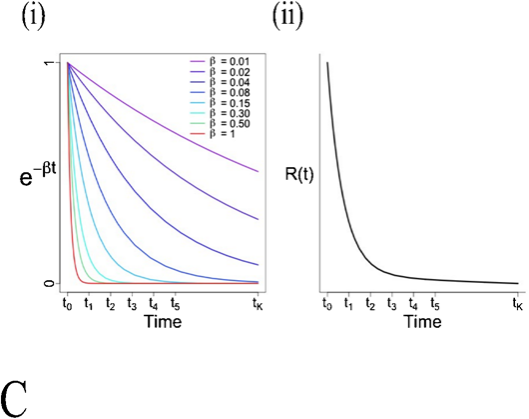	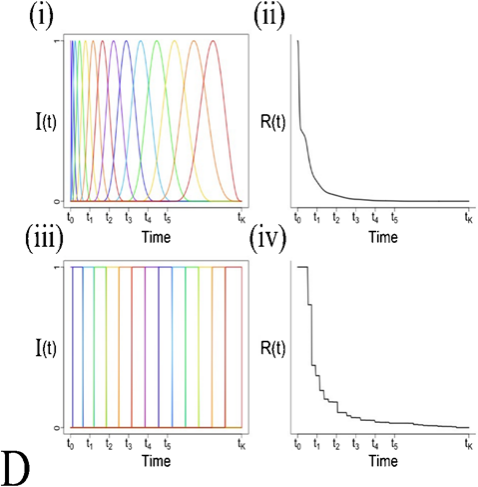
Formula	1C: $\frac{ d{ C }_{ 1 }(t) }{ dt } ={ K }_{ 1 }{ C }_{ p }(t)-{ k }_{ 2 }{ C }_{ 1 }(t)$			
	2C: $$ \left\{ \begin{array}{l} \frac{ d{ C }_{ 1 } }{ dt } ={ K }_{ 1 }{ C }_{ p }(t)-({ k }_{ 2 }+k_{ 3 }){ C }_{ 1 }(t)+{ k }_{ 4 }{ C }_{ 2 }(t) \\ \\ \frac{ d{ C }_{ 2 } }{ dt } ={ k }_{ 3 }{ C }_{ 1 }(t)-{ k }_{ 4 }{ C }_{ 2 }(t) \end{array} \right. $$	$\frac{ { C }_{ T }(t) }{ { C }_{ p }(t) } ={ K }_{ i }\frac{ \int _{ 0 }^{ t }{ { C }_{ p }(\tau )d\tau } }{ { C }_{ p }(t) } +C, t\ge { t }^{ * }$	${ C }_{ T}(t) = \sum _{ j=0 }^{ J }{ { \alpha }_{ j } g_j(t) },\alpha _j \ge 0$	${ C }_{ T}(t) = \sum _{ j=0 }^{ J }{ { \alpha }_{ j } f_j(t) }, \alpha _j \ge 0$
Residue form	1C: $R(t)={K}_{1}{ e }^{ -{ { k }_{ 2 }t } }$			
	2C: $R(t)={K}_{1}({ \pi }_{ 1 }{ e }^{ -{ \theta }_{ 1 }t }+{ \pi }_{ 2 }{ e }^{ -{ \theta }_{ 2 }t })$	$R(t)=K_i, t\ge t^*$	$R(t)=\sum _{ j=0 }^{ J }{ { \alpha }_{ j }{ e }^{ -{ \beta }_{ j }t } }$	$R(t)=\sum _{ j=1 }^{ J }{ { \alpha }_{ j }I_j(t) }$
Kinetic parameters	All	Only $K_i$	All	All
Computation	Nonlinear least square or maximum likelihood	Ordinary least square	Non-negative least square	Quadratic programming
Application to TB PET	Yes	Yes	No	Yes
References	[54, 57, 60, 63, 66, 68, 70, 72, 78, 82–84]	[31, 32, 43, 55, 57, 64, 70, 78, 86, 139]		[140]

*For details of derivations, see [88]

A: One (1C) and two compartmental (2C) models. $C_p$ is the plasma compartment; $C_1$ and $C_2$ are tissue compartments

B: Patlak plot

C: Spectral analysis. (i) exponential distribution for different values of $\beta$; (ii) the typical residue estimated by spectral analysis

D: Non-parametric analysis. (i) general form of B-spline basis elements; (ii) the typical residue estimated by B-spline function;

(iii) general form of piece-wise elements; (iv) the typical residue estimated by piece-wise function

Notations: $$\left\{ \begin{array}{lrr} { \pi }_{ 1 }=\frac{ { k }_{ 4 }-{ \theta }_{ 1 }+{ k }_{ 3 } }{ { \theta }_{ 2 }-{ \theta }_{ 1 } }, { \pi }_{ 2 }=\frac{ { \theta }_{ 2 }-{ k }_{ 4 }-{ k }_{ 3 } }{ { \theta }_{ 2 }-{ \theta }_{ 1 } } \\ \\ { \theta }_{ 1 }=\frac{ { k }_{ 2 }+{ k }_{ 3 }+{ k }_{ 4 }-\sqrt{ { ({ k }_{ 2 }+{ k }_{ 3 }+{ k }_{ 4 }) }^{ 2 }-4{ k }_{ 2 }{ k }_{ 4 } } }{ 2 } \\ \\ { \theta }_{ 2 }=\frac{ { k }_{ 2 }+{ k }_{ 3 }+{ k }_{ 4 }+\sqrt{ { ({ k }_{ 2 }+{ k }_{ 3 }+{ k }_{ 4 }) }^{ 2 }-4{ k }_{ 2 }{ k }_{ 4 } } }{ 2 } \end{array} \right. $$ $\quad g_j(t) = C_p(t) \otimes { e }^{ -{ \beta }_{ j }t }$ $\quad f_j(t) = C_p(t) \otimes I_j(t),$ *I* represents basis function (B-spline or piece-wise form)

## Kinetic model

Many kinetic models have been well-developed for quantitative PET scanning, but they differ in terms of residue form and produced information [49]. A summary is shown in Table 3. Most of them (e.g., compartmental model, Patlak plot, and spectral analysis) is a parametric model that generally relies on the necessary assumptions. These are difficult to justify for the heterogeneous tissue region, especially the diverse-tissue study. The non-parametric method without the assumption requirement should be more flexible and indeed have some substantial advantages.

Here, we provide an overview of various parametric and nonparametric strategies (see [88] for more details of derivations) and summarize their recent developments for total-body PET imaging [54, 64, 73, 78, 83, 84, 86, 139, 140]. The feasibility, challenge, and promise of these methodologies are also discussed.

### **Compartmental model**

Compartmental modeling forms the basis for tracer kinetics of dynamic PET data. There are two most important models used to derive physiological information in absolute measurement units as shown in Table 3 (A). One tissue compartmental (1C) model with two rate constants ($K_1$ in ml/min/cm${}^3$ and $k_2$ in min${}^{-1}$) was developed by Kety [141] for quantitative assessment of blood flow (perfusion). Two tissue reversible compartmental (2Cr) model with four rate constants ($K_1$ in ml/min/cm$^3$, $k_2$, $k_3$ and $k_4$ in min${}^{-1}$) is mainly used for quantifying receptor-ligand binding studies [142]. While $k_4$ equals 0 (irreversible), it becomes the most famous Sokolov-Huang model (2Ci) generally employed for the quantitation of metabolic rate for glucose [143–145]. For more generalized compartmental models and detailed underlying biochemical mechanisms, see [48].

These models are described by a system of first-order time-dependent differential equations, which can be solved by a numerical procedure known as nonlinear least squares (NLS) in order to appropriately estimate the residue function and associated kinetics. The advantages of compartmental modeling are the reliability and independency on the scanning time. When dealing with very noisy data (e.g., voxel-level analysis), this method has several shortcomings including convergence issues, long computational time, and sensitivity to initial estimates due to the nature of NLS [49].

#### 1C model

One tissue compartmental model is given by a differential equation as Eq. 5:

5 $$\begin{aligned} \frac{ d{ C }_{ 1 }(t) }{ dt } ={ K }_{ 1 }{ C }_{ p }(t)-{ k }_{ 2 }{ C }_{ 1 }(t) \end{aligned}$$

where $C_1$ represents tissue compartment and $C_p$ is the plasma compartment. Solved by the integrating factor method, the solution is found to be as follows:

6 $$\begin{aligned} { C }_{ 1 }(t)=\int _{ 0 }^{ t }{ { K }_{ 1 }{ e }^{ { -k }_{ 2 }(t-s) }{ C }_{ p }(s)ds } \end{aligned}$$

Related to the simple basic Eq. 1, the residue function can be expressed as

7 $$\begin{aligned} R(t)={K}_{1}{ e }^{ -{ { k }_{ 2 }t } } \end{aligned}$$

Hence, the parameter of interest - blood flow (perfusion) $=R(0)={K}_{1}$.

#### 2C model

Similar to the 1C model, two tissue compartmental model is represented by a coupled system of differential equations as Eq. 8.

8 $$\begin{aligned} \left\{ \begin{array}{lr} \frac{ d{ C }_{ 1 } }{ dt } ={ K }_{ 1 }{ C }_{ p }(t)-({ k }_{ 2 }+k_{ 3 }){ C }_{ 1 }(t)+{ k }_{ 4 }{ C }_{ 2 }(t) \\ \\ \frac{ d{ C }_{ 2 } }{ dt } ={ k }_{ 3 }{ C }_{ 1 }(t)-{ k }_{ 4 }{ C }_{ 2 }(t) \end{array} \right. \end{aligned}$$

where $C_2$ is the tissue compartment. By the Laplace transform and its inversion [146], the final result is given by the following:

9 $$\begin{aligned} C_{ T }(t)={ C }_{ 1 }(t)+{ C }_{ 2 }(t)={ K }_{ 1 }({ \pi }_{ 1 }{ e }^{ -{ \theta }_{ 1 }t }+{ \pi }_{ 2 }{ e }^{ -{ \theta }_{ 2 }t })\otimes { C }_{ p }(t) \end{aligned}$$

where

$$ \left\{ \begin{array}{lr} { \pi }_{ 1 }=\frac{ { k }_{ 4 }-{ \theta }_{ 1 }+{ k }_{ 3 } }{ { \theta }_{ 2 }-{ \theta }_{ 1 } }, { \pi }_{ 2 }=\frac{ { \theta }_{ 2 }-{ k }_{ 4 }-{ k }_{ 3 } }{ { \theta }_{ 2 }-{ \theta }_{ 1 } } \\ \\ { \theta }_{ 1 }=\frac{ { k }_{ 2 }+{ k }_{ 3 }+{ k }_{ 4 }-\sqrt{ { ({ k }_{ 2 }+{ k }_{ 3 }+{ k }_{ 4 }) }^{ 2 }-4{ k }_{ 2 }{ k }_{ 4 } } }{ 2 } \\ \\ { \theta }_{ 2 }=\frac{ { k }_{ 2 }+{ k }_{ 3 }+{ k }_{ 4 }+\sqrt{ { ({ k }_{ 2 }+{ k }_{ 3 }+{ k }_{ 4 }) }^{ 2 }-4{ k }_{ 2 }{ k }_{ 4 } } }{ 2 } \\ \end{array} \right. $$

Again recall the fundamental Eq. 1, residue is a mixture of exponentials as Eq. 10.

10 $$\begin{aligned} R(t)={K}_{1}({ \pi }_{ 1 }{ e }^{ -{ \theta }_{ 1 }t }+{ \pi }_{ 2 }{ e }^{ -{ \theta }_{ 2 }t }) \end{aligned}$$

For the irreversible 2C model ($k_4=0$), the metabolic flux is focused, that is $K_i= \underset{t\rightarrow \infty }{lim}R(t)=\frac{ { K }_{ 1 }{ k }_{ 3 } }{ { k }_{ 2 }+{ k }_{ 3 } }$. For the reversible tracers, volume of distribution is calculated as: $V_D=\int _{ 0 }^{ \infty }{ R(t)dt } =\frac{K_1}{k_2} (1+\frac{ { k }_{ 3 } }{ { k }_{ 4 } })$.

#### Delay effect

In the routine PET image, IDIF is usually extracted from a nearby arterial blood pool, so the time delay between IDIF and the targeted tissue is very short and even negligible. The total-body PET scanner provides several options for IDIF location that may be far away from some tissues. The delay time can be up to 50 s and significantly varied to different tissues, which has been an important factor to affect the kinetic quantification [54, 82–84].

To correct this effect, the delay term is jointly estimated with other parameters in compartmental models. Take 1C model as an example, replacing the residue function in Eq. 3 by Eq. 7 gives:

11 $$\begin{aligned} { C }_{ T }(t) \!=\! { V }_{ B }{ C }_{ p }(t-\Delta ) \!+\! (1-{ V }_{ B })\int _{ 0 }^{ t }{{K}_{1}{ e }^{ -{ { k }_{ 2 } (t-s) } } { C }_{ p }(s\!-\!\Delta )ds} \end{aligned}$$

In this setting, $(V_B,K_1,k2,\Delta )$ are estimated. Similarly for 2C model, $(V_B,K_1,k2,k3,\Delta )$ or $(V_B,K_1,k2,k3,k4,\Delta )$ are derived, but the estimation procedure is more computationally expensive. Two schemes have been proposed to determine the delay by only the first few minutes data using 1C model [82, 83] or full-time data in arbitrary models [54]. The former one has been initially demonstrated to be efficient [84].

#### Model selection

The selection of compartmental models (1C, 2Ci, 2Cr) usually depends on the tracer property, the aim of study, and even the organ or tissue of interest. For example, 1C is generally adopted for ${}^{15}$O-H${}_2$O and 2Cr is used for ${}^{11}$C-Raclopride. As the most commonly used tracer - ${}^{18}$F-FDG, the irreversible model (2Ci) is employed for many organs while its uptake into the liver exhibits reversible characteristics [147]. Therefore, we must justify each case carefully for the use of compartmental model.

The typical quantitation for dynamic PET study is applying a single model, which works well in organ-specific imaging on conventional scanners. But it may not be appropriate for total-body imaging as a single model is hard to be feasible for diverse tissues and organs. Wang et al. have reported that voxel-level model selection strategy based on an Akaike information criterion (AIC) leads to improved total-body parametric imaging [54]. But there is no doubt that it brings more computation burden. Later on, a further examination of various compartmental models for multiple organs is implemented at the ROI level [84]. This study indicates that the applicability of compartmental models for the bladder is questionable.

### Patlak plot

Graphical techniques provide simple ways to estimate the specific kinetic parameters by appropriately transforming the equations of compartmental modeling for irreversible and reversible tracers [148–150]. Here, we just focus on the most popular graphical method—Patlak model; for more details about other approaches, we suggest a review article for further reading [151]. Patlak analysis has been widely applied to dynamic PET imaging due to its simplicity and robustness [148], which is assumed that (i) the trapping of tracer in studied organs/tissues is completely irreversible; (ii) Patlak plot results in a straight line after the time that steady-state conditions between reversible tissue and plasma compartments are reached. If both assumptions are satisfied, $K_i$ can be estimated easily as the slope of Patlak plot after the equilibrium time ($t^*$) using linear regression. The Patlak plot is given by the expression below:

12 $$\begin{aligned} \frac{ { C }_{ T }(t) }{ { C }_{ p }(t) } ={ K }_{ i }\frac{ \int _{ 0 }^{ t }{ { C }_{ p }(\tau )d\tau } }{ { C }_{ p }(t) } + constant, \quad t\ge { t }^{ * } \end{aligned}$$

$K_i$ is computed using a few late time frames of dynamic scanning by a non-iterative strategy—ordinary least square (OLS). Due to the nature of linearity, it should be much faster and less sensitive to noise than NLS, and it is therefore appropriate for applications at the voxel level [8]. On the other hand, it must be noted that this approach does not provide any insight regarding the complete profile of tracer kinetics and only a reduced set of parameters ($K_i$) is obtained.

When adopting the standard Patlak (sPatlak) method for dynamic total-body imaging, many tissues and organs can be studied simultaneously. Single $t^*$ may not be appropriate for the diverse-tissue environment as the equilibrium conditions probably achieve at different time points. The feasibility of Patlak plot also needs to be justified for certain tissues like the liver, kidney, and bladder. These limitations and possible solutions are discussed in detail in the following.

#### Selection of t*

The improper $t^*$ may introduce additional errors in estimated $K_i$ [152]. A rich literature has explored the choice of $t^*$ for single organ on short AFOV PET scanners, for example, 20 min for brain [153] and 10 min for lung [154]. Total-body Patlak images are generated with various $t^*$, from 10 min [155], 15 min [29], 20 min [156] to 30 min [64]. But there are no more details about the justifications in these studies.

Recently, an adaptive $t^*$ scheme has been proposed to determine the optimal options for different ROIs or voxels [139]. It is based on two criteria: max-error and R squared ($R^2$). Max-error is defined as the worst case error between the predicted value and the true value for all observations on Patlak plot. The selected $t^*$ is the earliest one so that max-error is less than a threshold value. This criterion has been employed in PMOD ($Z\ddot{u}rich, Swizerland$), and the default setting of threshold is $10\%$. $R^2$ is a common metric to quantify the goodness of linear fit, and a value closer to 1 indicates a better fit, so optimal $t^*$ is determined by the maximum $R^2$. This procedure has the potential to improve the accuracy of kinetic parameters. However, further investigations in patient cohorts and more sophisticated techniques need to be developed.

#### Generalized patlak

As described above, the standard Patlak analysis assumes an irreversible 2C model. For total-body imaging, this assumption can be broken by some tissues (e.g., the liver where ${}^{18}$F-FDG may exhibit mild positive uptake reversibility and bladder associated with the complex tracer excretion process) [84, 139, 157] and tumors (e.g., hepatocellular carcinoma) [158] so that the sPatlak plot is no longer linear.

To address these issues, a generalized Patlak (gPatlak) method Eq. 13 based on the reversible 2C model was proposed in 1985 [159], which introduced an additional exponential term characterized by the net efflux ($k_{loss}$) to account for the effect of tracer dephosphorylation properly.

13 $$\begin{aligned} \frac{C_T(t)}{C_p(t)} = K_i \frac{\int _{0}^{t} e^{-k_{loss}(t-\tau )}C_p(\tau )d \tau }{ C_p(t)} + constant, t\ge { t }^{ * } \end{aligned}$$

This model becomes non-linear due to the added exponential term, but it can be solved by applying a basis function to linearize the estimation process [160].

The utility of gPatlak approach for diverse organs and tissues is first examined by Karakatsanis et al. [160] in multi-bed multi-pass whole-body PET imaging. Then, the performance of both standard and generalized Patlak methods has been assessed for multiple organs at the ROI level using a total-body PET study on uEXPLORER [86]. Results show that gPatlak can bring benefits for the liver, kidney, lung, and especially bladder. Thus, it would be also interesting to explore the use of gPatlak plot for voxel-level analysis in the future.

### Spectral analysis

The residue function in the compartmental model is the single exponential Eq. 7 or a mixture of exponentials Eq. 10. It may not have sufficient degrees of freedom to capture full variability in total-body PET data. Spectral analysis (SA) proposed by Cunningham and Jones in 1993 [161] assumes the residue to be the sum of $J+1$ exponential terms.

14 $$\begin{aligned} R(t)=\sum _{ j=0 }^{ J }{ { \alpha }_{ j }{ e }^{ -{ \beta }_{ j }t } }, \alpha _j \ge 0, \beta _j \ge 0, \beta _0=0 \end{aligned}$$

Thus, the tissue time course can be expressed as

15 $$\begin{aligned} { C }_{ T}(t) = \sum _{ j=0 }^{ J }{ { \alpha }_{ j }{ e }^{ -{ \beta }_{ j }t } } \otimes { C }_{ p }(t)\equiv \sum _{ j=0 }^{ J }{ { \alpha }_{ j } g_j(t) } \end{aligned}$$

$g_j(t)$ are known with the pre-defined eigenvalues $\beta _j$, whereas the amplitudes $\alpha _j$ are estimated by the NLS algorithm. The model structure (e.g., reversibility or irreversibility, number of compartments) is derived from $\alpha _j$, also called spectrum [8]. The information of macroparameters, such as *K*, $K_i$, and $V_D$ is obtained as follows:

16 $$\begin{aligned} K=\sum _{j=0}^{J}\alpha _j , \quad K_i=\alpha _0, \quad V_D=\sum _{j=1}^{J}\frac{\alpha _j}{\beta _j} \end{aligned}$$

Some relevant strategies such as rank-shaping spectral analysis [162] and spectral analysis with iterative filter [163] have also been developed in recent years. The main strength of spectral analysis is its flexibility which can be applied to reversible or irreversible tracers, single or multiple compartmental models, and homogeneous as well as heterogeneous systems [50]. These characteristics make this method adaptable to various tracers and particularly suitable for total-body PET imaging. But until now, it has not been implemented in this area.

### Non-parametric analysis

Typically, the tissue residue is a monotone decreasing function and approximated as nonnegative sums of exponential terms in the compartmental framework. However, the strict monotonicity ($\Delta R(t)<0$) is not always realistic[164] and the assumed exponential form may not be reasonable to represent data in which in vivo biochemistry is not clear [165–168], especially for the emerging total-body PET imaging [84, 86].

Unlike the methods discussed above, residue can be also estimated by the non-parametric approaches [169–172] and given by the following:

17 $$\begin{aligned} R(t)=\sum _{ j=1 }^{ J }{ { \alpha }_{ j }I_j(t) }, \quad \alpha _j \ge 0 \end{aligned}$$

Although it has a similar structure as Eq. 14, *I* here represents the basis elements, which can be B-spline [169, 172] or piece-wise function [170, 171]. This procedure has the ability to adapt to monotone (even exponential) and non-monotone forms as no unrealistic parametric restrictions are imposed.

The non-parametric residue analysis can be implemented rapidly by quadratic programming and has the advantage to provide more accurate kinetic quantitation in multiple tissues. An efficient application of this concept to generate parametric imaging is described as follows.

#### Non-parametric residue mapping

The non-parametric residue mapping (NPRM) consists of a fully automatic process incorporating data-adaptive segmentation, non-parametric residue analysis of segment data (sub-TAC), and voxel-level kinetic mapping scheme [173].

Following the linear structure of mixture model [93], the voxel-level time course ($z_i$) can be expressed as a non-negative combination of sub-TACs ($\mu _l$). The mechanism enables to address the heterogeneity of voxel-level data.

18 $$\begin{aligned} z_i(t)=\sum _{l=1}^{L}{\pi _{il}\mu _l(t)},\quad \pi _{il} \ge 0, \quad i=1,2,...,N \end{aligned}$$

where $\pi$ is the coefficient and *N* is the number of voxels.

For each sub-TAC, the associated residue is estimated non-parametrically, and the parameter of interest - $\theta$ (e.g., *K*, $K_i$ or $V_D$) can be derived as a function (*g*) of residue.

$$\begin{aligned} \mu _l(t)=R_l(t)\otimes { C }_{ p }(t-\Delta ) \Rightarrow \theta _l=g(R_l) \end{aligned}$$

The final parametric imaging is obtained as

19 $$\begin{aligned} \theta _i=\sum _{l=1}^{L}{\pi _{il}\theta _l}, \quad i=1,2,...,N \end{aligned}$$

The NPRM approach has some important features like the flexibility for diverse tissues and consideration of delays for different parts and also the ability to address issues with bladder or injection site [169, 171, 173], which make it feasible to be applied to total-body PET studies.

Building on Eq. 18, an image-domain bootstrap data generation process can be defined by the spatial and temporal patterns of model residuals [174, 175]. It has been used to assess the uncertainty (standard errors) of parametric imaging [176]. The practicality of simultaneous segmentation, kinetic parameter estimation, and uncertainty evaluation has also been demonstrated for a total-body breast cancer patient study on Biograph Vision Quadra [140].

## Other approaches

All the aforementioned approaches are applied in the image domain; however, they can be incorporated into the reconstruction process to estimate kinetic parameters by modeling projection data (sinogram or list-mode), known as the “direct method” [177]. The ideas for direct estimation could date back to the 1980s [178, 179], and since then, many scientists made great contributions to the progression of this technology for more accurate kinetics than the routine post-reconstruction procedure [180–185]. We suggest a detailed technical review for further reading [186]. It is remarkable that direct Patlak has been adopted on commercial scanners and applied to total-body PET studies [64, 78, 187]. But it suffers from similar problems like the non-linearity for specific tissues as mentioned above [86, 139, 188].

Another research interest in future work is the implementation of artificial intelligence (AI) for the total-body PET imaging [99, 189]. As a subcategory of AI, deep learning (DL) techniques, e.g., convolutional neural network (CNN) [190] and generative adversarial network (GAN) [101], have been extensively used in PET for solving a wide variety of problems involving image reconstruction [191–193], denoising [194, 195], segmentation [196, 197], and quantitation [198, 199]. A few initial attempts have been made to extract the flux ($K_i$) from total-body PET studies by DL methods [71, 187, 200]. More opportunities and challenges facing the adoption of DL in total-body PET quantitation are detailedly discussed in a recent review paper [85].

There are a number of PET studies where dynamic scans are used and main organs are included, e.g., whole-body human and preclinical animal imaging. The data structures and characteristics are similar to total-body human studies. Therefore, it is natural to generalize the techniques developed in these studies for quantifying dynamic total-body imaging. For example, (i) generalized and direct Patlak methods are both first examined for multiple organs in whole-body scans [160, 201, 202], then applied to total-body imaging [64, 78, 86]; (ii) the above-mentioned NPRM procedure is demonstrated in the whole-body pregnant macaque studies [203] before it is employed to generate total-body parametric imaging [140]. Many other perspectives also have excellent potential as tools in the future [22, 204].

## Discussion

Outside of the quantitative procedures discussed in this review, there are some basic challenges (e.g., motion, spillover, and partial volume) in the pre-processing strategy that may limit the reliability of estimated kinetics. Patient movement, respiration motion, and cardiac motion are unavoidable during the PET acquisition, particularly for the dynamic scanning with longer time. Many methods to correct motion have been proposed, and most of them are based on image registration algorithms or hardware motion tracking using an external device [49]. To the best of our knowledge, there is no common approach to resolve this issue for all organs even if it is well studied in the brain images. But we are glad to see that it has been investigated in the total-body studies by some researchers [205]. Another measure, denoising, is sometimes taken to ensure accurate results. Typically, one selected filter, e.g., Gaussian or non-local mean, is applied to reduce the PET image noise before the formal quantitation [206]. For a more comprehensive discussion on pre-processing procedures, we refer the reader to a recent article [87].

Although the emergence of total-body PET scanners brings a series of benefits, the concerns of the adoption of dynamic studies in clinical practice still remain, even more serious. For example, more static scans can be completed in a specific time interval (e.g.,1 h) as they can be acquired faster on uEXPLORER [30]. It may be argued that the cost of dynamic studies would be substantially higher. Thus, some protocol designs, e.g., dual-injection scheme [69], have been explored to reduce the dynamic scanning time. At the same time, parameter estimation procedures including non-invasive input functions and improved kinetic models are developed to make dynamic imaging more feasible and valuable in routine use [177]. Regardless of these difficulties, the additional information recovered from dynamic PET scans has been demonstrated to be useful to predict therapy response or survival [89, 90], which deserves to be appreciated in precision medicine for improving individualized treatment by maximizing the therapeutic effect and minimizing toxicity [46]. From all these perspectives, the role of dynamic PET imaging may not be changed in the short run, but we are confident that it must have a bright future in clinics.

During the past few years, many groups (>20) in nuclear medicine, physics, biomedical engineering, and statistics have been involved in the total-body PET data acquisition and analysis. The early adopters have generously shared their insights into this new technology. Hicks provided an installation guide including many aspects (e.g., financing, space, and power) for total-body PET/CT beginners [207]. Vandenberghe et al. proposed a few design options to reduce the cost for total-body PET [208]. Bern group shared their experience obtained from 7000 patient studies on Quadra [209]. An expert consensus was also proposed for the oncological use of uEXPLORER with $^{18}$F-FDG based on the experience of imaging 40,000 cases [210]. These reports greatly improve our understanding of the clinical use of advanced total-body systems. However, until now, these is no standardized framework for data structure, storage, sharing, and reproducibility, which may be similar to the Brain Imaging Data Structure (BIDS) platform promoted by the brain imaging community [211, 212]. The construction of such a platform needs the collaboration of multiple teams in diverse disciplines, but it is a worthwhile endeavor to release the full potential and pave the way for further developments of total-body PET scanners.

## Conclusions

In the coming years, total-body PET technologies are expected to have a more widespread impact. The review of basic principles and recent advances in general quantitation strategies may facilitate their use and validation in total-body imaging and subsequently enhance the reliability of derived kinetic information. The promise of some novel approaches (either deep learning or multivariate statistical methods) to improve quantitative accuracy is also pointed out. Overall, there is still a long way to fully understand and handle the complexities of total-body dynamics.
